# Key Predictive Factors in the Mental Health of Chinese University Students at Home and Abroad

**DOI:** 10.3390/ijerph192316103

**Published:** 2022-12-01

**Authors:** Jian Zhao, Elaine Chapman, Stephen Houghton

**Affiliations:** Graduate School of Education, The University of Western Australia, Crawley 6009, Australia

**Keywords:** mental health, higher education, personality traits, internal strengths, adverse life events, coping strategies, path analysis

## Abstract

The prevalence of reported mental health problems among university students has increased at alarming rates in recent years. While various negative life events (from personal events such as relationship breakdowns to more global events such as COVID-19 [SARS-CoV-2] pandemic) have been found to be important predictors of poor mental health in this population, some individuals have been found robustly to fare better than others in confronting such events. Identifying factors that predict these individuals’ mental health, along with the specific coping strategies they utilize may have significant practical implications when confronted by adverse events such as COVID-19. This study investigated relationships between the impact of the COVID-19 pandemic on 828 (453 females, 374 males, and one “Other”) Chinese university students’ mental health, and their internal strengths, personality characteristics, and demographic profiles. We also investigated whether students’ use of specific coping strategies mediated these relationships. Stepwise multiple regression analyses (MRAs) and a path analysis revealed that students who resided in their home country, had higher levels of internal strengths, a lower level of neuroticism and a higher level of agreeableness and reported fewer negative mental health changes than did other respondents during COVID-19 in the second half of 2020. Self-regulation and withdrawal coping strategies were both important mediators of these relationships. These findings have important implications for universities in identifying and assisting students in the face of adverse events such as COVID-19.

## 1. Introduction

### 1.1. Background

Positive mental health is critical to the academic success of university students [1]. A large body of research has now shown, however, that university students are at high risk of developing symptoms of poor mental health (including stress, anxiety, and depression) over the course of the studies by Mofatteh [2] and Pedrelli [3]. Mental health problems such as depression and anxiety have been found to reduce students’ motivation and concentration, and concurrently increase their mental fatigue levels [4,5,6]. This would not only reduce students’ academic performance and overall quality of life, but also increase the burden of care carried by university counselling services, many of which are ill-equipped to cope with these demands [7,8,9]. 

Mental health issues may result from a constellation of factors, including both genetic predispositions [10,11] and environmental or situational variables [12]. Exposure to adverse life events, or events that are “undesirable, uncontrollable, and unpredictable” [13] (p. 187), has also been shown to be an important predictor of poor mental health [14,15]. Adverse life events typically include personal events such as divorce or serious relationship breakdowns, death of loved ones, disease, bankruptcy and unemployment, but may also include more global events such as natural disasters, world wars, and global pandemics such as Ebola and COVID-19 [16,17,18,19,20,21,22,23].

In a meta-analysis published almost two decades ago, Kraaij et al. [24] reviewed 25 studies that had explored relationships between adverse life events (e.g., death of significant others, severe illness of self or significant others, financial problems, disasters, and crimes) and depression amongst elderly people. They found that all such events related significantly to depression in this population. Research conducted with other populations since that time has produced similar results. For example, Hassanzadeh et al. [25] reported that stressful life events (e.g., divorce or separation, financial problems, personal conflicts) were significantly related to reported psychological problems in 4736 Iranian adults. Ji et al. [26] found that adverse life events (e.g., academic stress, loss, health and adaptation problems) were significantly related to depressive symptoms and poor academic engagement amongst 3629 Chinese university students. All such evidence underscores, in diverse populations, the potential negative impact of adverse events on mental health.

### 1.2. Predictors of the Impact of Adverse Life Events on Mental Health

While extensive evidence has now been amassed on the links between adverse life events and poor mental health, research has indicated that some people robustly fare better than others in confronting such events [27]. If the factors that predict the degree of the negative impact that adverse life events will have on individuals can be identified, practitioners may be better placed to identify which individuals are likely to be more ‘at risk’ in these situations. This would, in turn, facilitate the provision of timely interventions and additional support for individuals at such risk. Aside from more ‘fixed’ factors such as genetic profiles [28], research has suggested four types of factors (i.e., problem-solving self-efficacy [29]; internal locus of control [30]; ability to deal with uncertainty [31]; and mindfulness [32]), that may act as predictors of individuals’ responses to negative events in a range of populations.

#### 1.2.1. Internal Strengths

Research has suggested that individuals’ internal strengths, which include high problem-solving self-efficacy [29], internal locus of control [30], ability to deal with uncertainty [31] and mindfulness [32] may help to ‘buffer’ the impact of adverse events on individuals’ mental health. According to Zhao et al. [33], internal strengths can be an important antecedent in one’s overall adaptive response to negative life events. In particular, people with higher levels of internal strength are more likely to interpret an adverse event or experience as less threatening than those with lower levels of internal strength, and may in turn use more adaptive coping strategies to deal with this event. In contrast, those with relatively low levels of such attributes may quickly become overwhelmed in the face of such events, and choose more reactive, maladaptive coping strategies (e.g., withdrawal) to deal with their attendant effects. This may, in turn, have a negative long-term impact on mental health.

In a study of the relationship between internal strengths and mental health, Zhao et al. [33] identified clusters of individuals with high or low internal strength profiles (determined by levels of problem-solving self-efficacy, internal locus of control, ability to deal with uncertainty, and mindfulness), and explored whether mental wellbeing differed significantly across these internal strength clusters. Results also showed significant differences in the mental health and wellbeing of the two groups in the context of the COVID-19 pandemic, as measured by the WHO-5 [34] and the 12-item DASS [35]. Therefore, these four factors may be important moderators of the relationship between adverse life events and mental health.

#### 1.2.2. Personality Traits

Previous research has also suggested important links between personality variables and individuals’ ability to maintain their positive mental health in the face of negative life events. Personality traits are defined as “relatively enduring patterns of thoughts, feelings, and behaviors that distinguish individuals from one another” [36] (p. 31). Personality research today is dominated by the Five Factor Model [37,38,39,40], alternatively labelled the “Big Five” model [41]. The five dimensions in this model are Neuroticism (“a tendency to experience dysphoric affect-sadness, hopelessness, guilt”), Extraversion (“a preference for companionship and social stimulation”), Openness (“a need for variety, novelty and change”), Agreeableness (“a willingness to defer to others during interpersonal conflict”) and Conscientiousness (“a strong sense of purpose and high aspiration levels”). This model of personality is well validated, having emerged in factor analytic studies in all cultures in which it has thus far been studied [40] (p. 164).

Oshio et al. [42] reviewed 30 studies that had investigated relationships between resilience and Big Five personality traits, and found that neuroticism was negatively correlated with resilience, while all other traits were positively correlated with resilience. Fayombo [43] similarly investigated relationships between the Big Five personality traits and psychological resilience in Caribbean adolescents, and reported that psychological resilience was negatively correlated with neuroticism, while being correlated positively with conscientiousness, agreeableness, and openness to new experiences. Therefore, whilst the personality traits of extraversion, openness to new experiences, agreeableness and conscientiousness have less often been studied than neuroticism with respect to the impact of adverse events on mental health, studies have also suggested these variables may contribute positively to individuals’ ability to deal with such events [43,44,45].

Neuroticism has received particular attention in the literature on how individuals cope with negative life events. People with high levels of neuroticism tend to interpret ordinary situations as threatening and respond worse to stressors in terms of their anxiety levels [46,47]. Several studies conducted during COVID-19 have reported that low levels of neuroticism predicted better responses to the pandemic in terms of individuals’ resilience levels [48]. High levels of neuroticism, conversely, have been found to predict poor mental health in the face of such adversities [46,49]. Neuroticism, therefore, may also be an important negative predictor of individuals’ responses to adverse life events, including more global events such as the COVID-19 pandemic.

#### 1.2.3. Demographic Profiles

Previous research has also highlighted specific demographic factors as potential moderators of individuals’ responses to adverse events. For example, Yan et al. [50] found females were more vulnerable to developing mental health problems in response to the COVID-19 pandemic than males. Amongst potential explanations related to this effect, it is possible that women experienced more dramatic increases in stress during their day-to-day routines than did men, given that many children were forced to stay at home rather than attend school in this period, which may increase the care burden at home on women. It is also possible that reducing the availability of face-to-face interactions would have impacted women more than men during this time.

Jace and Makridis [51] also reported that married people were less likely to experience mental health problems during COVID-19 than their unmarried counterparts. This finding was supported by Nkire et al. [52], who reported that being single, separated or divorced were risk factors for developing more severe stress, anxiety, and depression during the COVID-19 pandemic. Based on these results, Nkire et al. [52] recommended that “Services aimed at providing mental health supports during pandemics should consider allocating more resources to supporting these particular groups of people” (p. 1). It may be that owing to the restrictions that the pandemic inevitably imposed, having ready access to a spouse helped to mitigate the impact of attendant lockdowns on loneliness and a sense of belongingness.

Another demographic variable that could affect how individuals respond to adverse events is the environment in which the events are confronted. For example, Zhao et al. [53] found that Chinese students in Chinese universities had better mental health than Chinese international students studying in Australian universities during COVID-19. In another study, very high levels of distress and perceived stress, in addition to low levels of wellbeing, were found amongst individuals who were affected by the COVID-19 border closures in Australia [54]. Therefore, being at home or abroad may also be an important moderator of how individuals respond to adverse events.

#### 1.2.4. Coping Strategies

Coping strategies are cognitive, emotional and/or behavioral strategies that people use in response to stressors [55]. It has been found that individuals who use different coping strategies when confronting adverse life events may experience varying degrees of mental health effects in response to these events. For example, Yu et al. [56] found that active coping strategies such as “identifying several different ways to solve problems” were negatively related to the psychological distress experienced by Chinese adults during COVID-19. AlHadi et al. [57] also found that denial and self-blame coping strategies were associated with more severe negative mental health responses to the COVID-19 pandemic in Saudi Arabian adults. Very recently, Zhao et al. [53] found that problem-solving and disengagement strategies were positively related to Chinese university students’ overall well-being during COVID-19. Given the associations that have been identified between coping strategies and mental health, it is plausible to assume that individuals who tend to use coping strategies that have been identified as more maladaptive (e.g., withdrawal) are more likely to be affected negatively by adverse life events, while those who tend to adopt strategies deemed to be more adaptive (e.g., problem-solving coping strategies) are likely to fare better in such situations.

### 1.3. The Present Study

Previous research suggests the impact of adverse life events on individuals’ mental health may be moderated by a number of factors, including specific demographic variables, personality traits, internal strengths, and coping strategies. The present study explored the relationship between all of these factors on Chinese university students’ mental health against the backdrop of the COVID-19 pandemic. Two specific research questions were addressed within the study:

To what extent did internal strengths, personality traits and demographic factors (marital status, sex, and country of residence) predict Chinese university students’ responses to the COVID-19 pandemic?

To what extent were the relationships between these variables mediated by the different coping strategies that Chinese university students used in dealing with the pandemic?

If factors that predict individuals’ mental health can be identified, and it is found that specific coping strategies mediate these relationships, this may have significant practical implications. In particular, given that coping strategies are ‘learnable’, students who are more ‘at risk’ when facing an adverse life event could be provided with additional support for confronting negative events which incorporates building their capacity to adopt more adaptive coping strategies. Universities may then draw upon this knowledge in devising appropriate counselling services for students in the face of broader adverse events such as the COVID-19 pandemic.

## 2. Method

### 2.1. Sampling

The study was based on a survey completed by Chinese students in late 2020/early 2021, who were studying either at Chinese or Australian universities. Participants completed the survey in response to an open invitation posted on Wechat. In total, 828 responses (453 females, 374 males, and one “Other”) were retained for analysis after removing incomplete and clearly disengaged responses. Of these, 732 were enrolled in universities across mainland China, while 96 were enrolled in an Australian university. The mean age of the group was 22.52 years (*SD* = 4.29). All participants completed the survey on a voluntary basis without any financial incentives.

### 2.2. Instruments

#### 2.2.1. The Internal Strengths for Adverse Life Events Scale (ISALES)

The ISALES [33] was used to measure four internal attributes that have been found to protect against the impact of adverse life events on mental health: (a) Problem-solving self-efficacy; (b) Internal locus of control; (c) Ability to deal with uncertainty; and (d) Mindfulness. This instrument comprises 16 items (four items per internal strength), all presented in bipolar statement format. Respondents rate each item on a 7-point scale (ranging from −3 to +3), with higher scores indicating higher levels of the relevant attribute. In a preliminary evaluation of the psychometric properties of this instrument, Zhao et al. [33] found that the ISALES exhibited excellent reliability and validity in this population.

#### 2.2.2. Mental Health Changes Indicators Scale (MHCIS)

The Chinese version of the MHCIS [50] was used as the outcome variable in the present study. The MHCIS is a 10-item self-report instrument developed by Zhao et al. [53] to measure the negative impact of nominated adverse events on individuals’ mental health. Each item captures changes in one specific negative indicator of mental health. Participants respond on a 5-point scale, ranging from 1 (no change) to 5 (extreme change). Total scores range from 10 to 50, with higher scores indicating a more negative impact of the event. Again, in a preliminary evaluation of this instrument, Zhao et al. [53] found that the MHCIS demonstrated excellent reliability and validity for use with Chinese university students.

#### 2.2.3. Coping Strategies Scale (CSS)

The Chinese version of the CSS [58] was used to measure the coping strategies participants chose to adopt in the COVID-19 context. The CSS includes 30 specific strategies which can be categorized into seven general types of coping strategies: Withdrawal, Positive Adaptation, Problem-solving, Disengagement, Prosocial Focus, Seeking Emotional Support, and Self-regulation. Respondents were asked to rate how frequently they used each of the strategies using a five-point scale (Never = 1, Rarely = 2, Sometimes = 3, Often = 4, Always = 5). Zhao et al. [58] have found that this instrument has sound internal structure and reliability in Chinese university populations.

#### 2.2.4. The Brief Big-5 Personality Inventory (BBPI)

The BBPI was used in this study to measure personality traits based on the five-factor model. The 15-item BBPI included three items to measure each of the ‘Big Five’ factors (conscientiousness, agreeableness, neuroticism, extraversion and openness). Each item was presented in bipolar statement format. Respondents indicated their responses on a scale from 1 to 7, as shown in Table 1. While this scale has not yet been validated, it was based directly on Lim & Chapman’s previous Big Five instrument [59], which has been validated in Chinese populations. The items were also closely aligned with other five-factor personality instruments in the field [39,60].

### 2.3. Data Analysis

To address Research Question 1, two stepwise multiple regression analyses (MRAs) were conducted to investigate whether demographic variables (sex, marital status, country of residence) and personality traits (conscientiousness, agreeableness, neuroticism, extraversion and openness) significantly predicted changes in participants’ mental health while confronting COVID-19. In the first MRA, demographic variables (sex, marital status and country of residence) were entered as independent variables and scores on the MHCIS were entered as the dependent variable. In the second MRA, the five personality traits were entered as independent variables, with MHCIS scores as the dependent variable. A *t*-test was then performed to compare participants in the high and low internal strength profile clusters.

To address Research Question 2, a path analysis was performed to examine the extent to which any significant relationships identified in the MRAs were mediated by the specific coping strategies that individuals used (Research Question 2). The MRAs were conducted using IBM SPSS V27, while the path analysis was conducted using LISREL V10.0.

## 3. Results

Prior to conducting any analysis, data screening was performed to ensure that all relevant assumptions were met for multiple regression and path analysis. All such analyses produced satisfactory results in terms of linearity, multicollinearity, independence, homoscedasticity, and multivariate normality. Results are presented here in line with the research questions posed.

### 3.1. Research Question 1

Descriptive statistics and correlations for all variables are shown in Table 2, and the outcomes of the MRAs are shown in Table 3.

The first MRA model indicated that there was only one significant predictor of MHCIS scores (i.e., country of residence), R^2^ = 0.03, *F*(1, 826) = 23.83, *p* < 0.001. The second multiple regression model indicated two personality traits (i.e., Neuroticism and Agreeableness) that significantly predicted MHCIS scores, R^2^ = 0.06, *F*(1, 825) = 7.20, *p* = 0.007. Other information on MRAs results can be seen in Table 3.

The positive correlation (0.17) between the country of residence (1 = China, 2 = Australia) and MHCIS indicated that students who resided in Australia tended to experience more significant negative mental health changes than their counterparts in China. The positive correlation (0.22) between Neuroticism and MHCIS scores, and the negative correlation (−0.17) between Agreeableness and MHCIS, indicated that students with higher levels of Neuroticism and lower levels of Agreeableness also tended to experience more significant negative mental health changes than other participants. 

The Welch test indicated that participants in the lower strengths profile group (*n* = 491, *SD* = 0.93) demonstrated significantly more negative mental health changes than those in the higher internal strength profile group (*n* = 337, *SD* = 0.74), *F*(1, 826) = 41.62, *p* < 0.001. As such, internal strength profiles also significantly predicted students’ mental health amidst COVID-19, which aligns with previous results reported by Zhao et al. [33].

### 3.2. Research Question 2

To explore the extent to which relationships between people’s internal strengths and their mental health changes (as measured by the MHCIS) were mediated by the adoption of different coping strategies, a path analysis was performed with three groups of variables. Group 1 included country of residence (China vs. Australia), Neuroticism, Agreeableness and internal strength profile, all of which emerged as significant predictors of students’ MHCIS scores in the previous MRAs. Group 2 (mediating variables) included seven categories of coping strategies (Positive Adaptation, Prosocial, Seeking Emotional Support and Self-regulation, Withdrawal, and Disengagement). Group 3 comprised one variable—MHCIS scores. 

**Figure 1:**
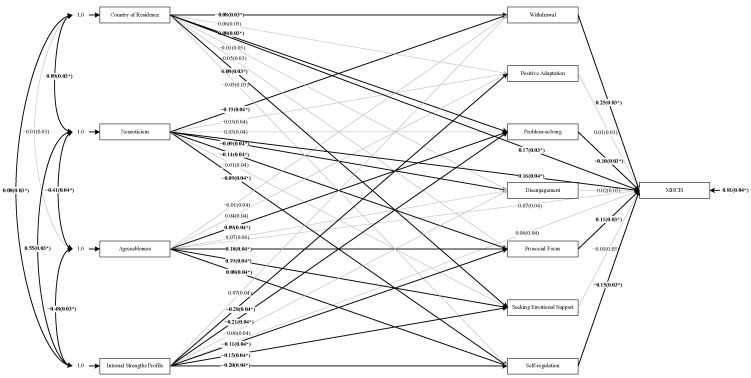
Path Analysis Diagram. “*” represents that direct effect between the two variables is significant at 0.05 level.

The outcomes of the path analysis, including the path coefficients associated with all direct effects in the model, are shown in Figure 1. According to Hoyle [61], *t*-values that fall beyond ±1.96 and ±2.56 are considered significant at the 0.05 and 0.01 levels, respectively. As indicated, the direct effects from the country of residence to withdrawal, problem-solving, and seeking emotional support, from neuroticism to withdrawal, disengagement, prosocial focus and self-regulation, from agreeableness to problem-solving, prosocial focus, seeking emotional support and self-regulation, as well as from cluster membership to all coping strategies (except for withdrawal and disengagement) are all significant. Direct effects from both the country of residence and neuroticism on MHCIS are significant.

All total indirect effects between Country of Residence, Neuroticism and Internal Strengths Profile and MHCIS scores were significant at the 0.01 level. The total indirect effect for Agreeableness was not significant.

Decomposition of the significant indirect effects indicated that the net indirect effect of the country of residence was negative. Five of the specific indirect effects were negative (via Withdrawal, Problem-Solving, Disengagement, Prosocial Focus, and Seeking Emotional Support), and the other two were positive (Positive Adaptation and Self-Regulation). Of the negative indirect effects (which together summed to −0.03), Withdrawal was the most prominent mediating variable, accounting for 56.31% of this effect. Of the positive indirect effects (which together, summed to only 0.008), the most prominent contributor was Self-regulation, which accounted for 92.16% of this effect. 

For Neuroticism, the net indirect effect was also negative. Four of the specific indirect effects were negative (via Withdrawal, Positive Adaptation, Disengagement, and Prosocial Focus), and the other three were positive (Problem-Solving, Self-Regulation and Seeking Emotional Support). Of the negative indirect effects (which together summed to −0.06), Withdrawal was the most prominent mediating variable, accounting for 73.49% of this effect. Of the positive indirect effects (which together, summed to only 0.005), the most prominent contributor was Problem-solving, which accounted for 56.78% of this effect.

Finally, for Internal Strength Profile, the net indirect effect was positive. Three of the specific indirect effects were negative (via Positive Adaptation, Disengagement, and Prosocial Focus), and the other four were positive (Withdrawal, Problem-Solving, Self-Regulation and Seeking Emotional Support). Of the negative indirect effects (which together summed to −0.002), Prosocial focus was the most prominent mediating variable, accounting for 75.27% of this effect. Of the positive indirect effects (which together, summed to 0.07), the most prominent contributors were Self-Regulation and Problem-Solving, which accounted for 43.85% and 34.30% of this effect (respectively).

## 4. Discussion

The present study aimed to explore the extent to which internal strengths, personality traits and demographic variables predicted changes in Chinese university students’ mental health during COVID-19, and also, the extent to which such relationships were mediated by the different coping strategies that students used in dealing with COVID-19. As indicated by the results of MRAs and the *t*-test, country of residence, neuroticism, agreeableness and internal strengths were all significant predictors of students’ mental health changes during COVID-19. More specifically, students who resided in China, had lower levels of neuroticism, higher levels of agreeableness and higher internal strengths reported fewer negative changes to their mental health than did those who resided in Australia, who had higher levels of neuroticism, lower levels of agreeableness, and lower internal strengths.

These findings are intuitively reasonable. First, compared with Chinese students who studied in their own country, those who studied abroad in Australia during that time may have been more vulnerable to mental health issues because they were in an unfamiliar context. International students who left their home country to live in a foreign country with an unfamiliar culture are likely to confront various challenges and difficulties [62]. In a report on the mental health of international students, Forbes-Mewett [63] (p. 5) pointed out that “nerves, confusion, depression, homesickness, loneliness, stress and insomnia” are all related to “being in an unfamiliar environment”. The different languages, different academic practices and different therapeutic approaches to ‘emotional’ issues between the home country and the host country [64] may all contribute to the challenges and difficulties while studying and living in a host country. Chinese international students who are studying in Australia may also potentially experience more discrimination than their counterparts studying in their home country, which might have been exacerbated during the COVID-19 period [53]. 

Second, people with high levels of neuroticism may have a greater tendency to interpret ordinary situations as threatening and thus respond more negatively to such stressors [46,47]. Several studies have reported that high levels of neuroticism predicted poor mental health in the face of COVID-19 [46]. Thus, these results align well with those from other studies in the field.

Third, individuals with higher levels of agreeableness are defined by a tendency to be more cooperative, tolerant and adaptive [65,66] than those with lower levels of agreeableness. This group, therefore, may adjust to the uncertainties and stress brought by COVID-19 more quickly and effectively. Proto and Zhang [67] also found that people who have higher levels of agreeableness have better mental health during COVID-19.

As shown by the path analysis in the present study, the coping strategies of self-regulation and withdrawal were the most prominent mediators of indirect relationships between the factors described above and MHCIS scores. In particular, Chinese students who resided in Australia tended to experience larger mental health changes (worse mental health outcomes). Meanwhile, these students were less likely to use withdrawal as a coping strategy, which was positively related to MHCIS; and more likely to use problem-solving as a coping strategy and seeking emotional support as a coping strategy, which was related negatively to MHCIS (worse mental health outcomes). Students who have higher neuroticism are less likely to use any coping strategies than those with lower neuroticism, and therefore more likely to experience more mental health changes (worse mental health outcomes). Students who have high internal strengths are more likely to use positive adaptation, problem-solving, prosocial focus, seeking emotional support and self-regulation than those with low internal strengths, and as a result, tend to experience fewer mental health changes (better mental health outcomes).

These findings are generally consistent with findings reported in previous literature. For example, according to Fluharty and Fancourt [68], avoidance coping (named withdrawal coping strategies in the current study) may help reduce short-term stress but may be harmful in the long-term as no direct actions are taken to reduce the stressor. This may lead to individuals feeling helpless in the long run. Adverse life events like COVID-19 not only brought immediate concerns such as food shortages due to lockdowns, but also long-term stressors such as negative effects on academic study, employment and financial situations. All of these may negatively influence people’s mental health. Since these issues cannot be addressed by the actions of individuals, the use of emotional self-regulation may be important to the maintenance of positive mental health. This has been confirmed by Ding et al. [69], who studied the impact of different coping strategies used by Chinese college students during COVID by examining the mediating role of perceived stress. Their findings suggested that when experiencing *uncontrollable* events such as COVID-19, focusing on positive ways to manage one’s own emotions can help reduce perceived stress, which will in turn produce lower levels of psychological distress. As a result, it is understandable that problem-solving coping strategies did not emerge as a prominent mediator in the present study, which focused upon COVID-19 as an uncontrollable event (i.e., was not something that individuals could ‘solve’ in any way). 

The results of the present study may have significant practical implications. Most importantly, universities may be able to use these results to develop mental health profiles of students, which help identify students more ‘at risk’ in the face of widespread adverse events such as COVID-19. This will not only allow such institutions to provide timely support for students who are most in need, but also, optimize the allocation of mental health services and resources. The results of this study, therefore, may have significant practical utility for all professionals charged with the provision of care for individuals in these situations.

## 5. Limitations

This study was not without limitations. First, as noted, the sample sizes for the two groups were highly discrepant, with a far higher number of Chinese students residing in China participating in the study. This was not avoidable due to the Australian border restrictions that were imposed in 2020 and 2021. The number of Chinese students studying in Australia was still sufficient to ensure the stability of the statistical estimates. Future studies, however, in different population groups (e.g., those from other countries or outside of universities), would be useful to confirm the generality of these results across contexts. Second, the variables included in this paper do not represent an exhaustive list of all variables that may affect students’ mental health. Future research could expand upon the existing research by including also variables such as gender, financial situation, health behaviors and chronic diseases, which have all been found to relate to individuals’ mental health [70,71,72,73]. Third, it was not possible to incorporate more specific location information of the specific location of Chinese students studying in China (e.g., province, city, whether from a rural area or urban area) in the study. This could also be addressed in a future study, because different amounts of resources and support may be available to those in different areas. Finally, given the non-experimental design of this study, no causal relationships between the predictors studied and participants’ mental health can be drawn at this point. Therefore, a longitudinal study, in which measures are taken at more regular intervals (e.g., monthly) over an extended period, would allow us to explore the progression trajectories of mental health outcomes for these students.

## Figures and Tables

**Table 1:** Items and Statements for BBPI-5.

Label	Low Scoring Statement (Scored 1 at Endpoint)	High Scoring Statement (Scored 7 at Endpoint)
C1	I tend to put in just enough effort to finish necessary tasks.	I put in extra effort to do all tasks thoroughly.
C2	Whenever I have something to do, I’ll try to minimise the time required to do it.	Whenever I have something to do, I make sure I take the time to do everything that is required.
C3	People who know me might consider me a bit lazy.	I would be seen as extremely conscientious by people who know me.
A1	Whenever I’m in a group, I will push for a certain outcome, regardless of how this might make others feel.	When I’m in a group, I consider it more important to make sure everyone is happy with the decisions made.
A2	I prefer to do things my way.	I prefer to work with other people to get things done.
A3	I don’t really care if people are comfortable with me.	It is extremely important to me that people feel comfortable with me.
N1	I tend to worry unnecessarily.	I’m not a person who tends to worry about things.
N2	I typically feel very stressed in difficult situations.	I am generally calm in difficult situations.
N3	I am a person who tends to worry about things that might happen.	I rarely worry about things that haven’t happened yet.
E1	I generally prefer to avoid large gatherings.	I really enjoy socialising in large groups.
E2	I would rather spend my time alone, doing my own things.	I consider myself a very sociable person.
E3	I consider myself to be more introverted than extroverted.	I consider myself to be more extroverted than introverted.
O1	Given the choice, I tend to prefer doing things I’m familiar with and enjoy rather than trying something new.	Given the choice, I will always want to try something new.
O2	I prefer it when things in my life stay pretty constant.	If possible, I would live a life full of surprises and new experiences.
O3	I prefer to stick with routines in general.	I prefer to do things differently all the time.

**Table 2:** Descriptive Statistics and Correlations for All Variables in the MRA (*n* = 828).

Factors	Descriptive Statistics		Correlations																				
	Mean	*SD*	1	2	3	4	5	6	7	8	9	10	11	12	13	14	15	16	17	18	19	20	21
1-Sex	1.45	0.50	1	0.05	−0.05	0.04	0.02	−0.14 **	0.05	0.01	0.09 *	−0.05	0.04	0.05	0.04	−0.08 *	0.02	0.05	0	0.06	0.08 *	−0.04	0.01
2-Marital status	1.73	0.44		1	−0.08 *	−0.11 **	−0.03	0.03	−0.04	−0.04	0.07 *	−0.11 **	−0.12 **	−0.01	0.04	−0.09 *	−0.09 **	−0.09 *	−0.08 *	−0.03	0	0.07 *	0.05
3-Country of residence	1.12	0.32			1	−0.08 *	−0.01	0.09 *	−0.08 *	−0.07	−0.09 *	0.03	0.06	−0.02	−0.08 *	0.070 *	−0.07 *	−0.05	−0.04	−0.13 **	−0.11 **	0.08 *	0.67 **
4-Personality_C	4.89	1.39				1	0.56 **	−0.46 **	0.42 **	0.50 **	0.03	0.27 **	0.29 **	0.09 **	0.30 **	0.24 **	0.28 **	0.56 **	0.57 **	0.47 **	0.54 **	−0.55 **	−0.15 **
5-Personality_A	5.02	1.19					1	−0.41 **	0.48 **	0.46 **	0.03	0.18 **	0.20 **	0.13 **	0.29 **	0.26 **	0.22 **	0.43 **	0.51 **	0.41 **	0.44 **	−0.48 **	−0.17 **
6-Personality_N	3.72	1.50						1	−0.61 **	−0.51 **	−0.15 **	−0.19 **	−0.18 **	−0.15 **	−0.27 **	−0.16 **	−0.24 **	−0.55 **	−0.44 **	−0.67 **	−0.56 **	0.55 **	0.22 **
7-Personality_E	4.02	1.65							1	0.59 **	0.18 **	0.13 **	0.13 **	0.13 **	0.34 **	0.20 **	0.23 **	0.45 **	0.38 **	0.53 **	0.56 **	−0.49 **	−0.13 **
8-Personality_O	4.68	1.49								1	0.07 *	0.28 **	0.26 **	0.14 **	0.30 **	0.22 **	0.28 **	0.55 **	0.50 **	0.56 **	0.55 **	−0.53 **	−0.10 **
9-C_Withdraw	2.43	0.88									1	0.03	0.04	0.39 **	0.39 **	0.13 **	0.10 **	−0.01	0	0.09 *	0.17 **	−0.04	0.24 **
10-C_PosiAdap	3.23	0.71										1	0.57 **	0.29 **	0.36 **	0.42 **	0.52 **	0.37 **	0.34 **	0.24 **	0.26 **	−0.31 **	−0.13 **
11-C_ProbSolv	3.38	0.73											1	0.27 **	0.42 **	0.51 **	0.51 **	0.33 **	0.35 **	0.21 **	0.24 **	−0.27 **	−0.16 **
12-C_Disengage	3.15	0.78												1	0.34 **	0.33 **	0.43 **	0.15 **	0.14 **	0.17 **	0.15 **	−0.14 **	0.02
13-C_Prosocial	2.92	0.85													1	0.40 **	0.43 **	0.29 **	0.25 **	0.29 **	0.34 **	−0.27 **	0.02
14-C_EmoSupp	3.31	0.82														1	0.49 **	0.29 **	0.29 **	0.22 **	0.24 **	−0.23 **	−0.09 *
15-C_Selfregu	3.37	0.80															1	0.39 **	0.35 **	0.29 **	0.27 **	−0.30 **	−0.20 **
16-Self-efficacy	4.96	1.39																1	0.58 **	0.60 **	0.53 **	−0.63 **	−0.24 **
17-Locus of control	5.01	1.15																	1	0.56 **	0.55 **	−0.66 **	−0.20 **
18-Dealwith uncert	4.54	1.40																		1	0.63 **	−0.66 **	−0.26 **
19-Mindfulness	4.58	1.24																			1	−0.70 **	−0.17 **
20-Clustermember	1.59	0.49																				1	0.22 **
21-MHCIS	1.82	0.88																					1

** Correlation is significant at the 0.01 level (2-tailed). * Correlation is significant at the 0.05 level (2-tailed).

**Table 3:** Stepwise MRAs Results (*n* = 828).

Variable	Mean	*SD*	Correlation with MHCIS	Multiple Regression Weights	
				b	β
MHCIS	1.82	0.88	-		
Country of residence	1.12	0.32	0.17	0.46	0.17
Neuroticism	3.72	1.50	0.22	0.10	0.18
Agreeableness	5.02	1.19	−0.17	−0.07	−0.10

## Data Availability

The data presented in this study are available on request from the corresponding author. The data are not publicly available due to ethical issues.
